# The “sowing of concrete”: Peri-urban smallholder perceptions of rural–urban land change in the Central Peruvian Andes^☆^

**DOI:** 10.1016/j.landusepol.2013.11.010

**Published:** 2014-05

**Authors:** Andreas Haller

**Affiliations:** Institute of Geography, University of Innsbruck, Innrain 52f, 6020 Innsbruck, Austria

**Keywords:** Environmental perception, Peri-urban growth, Landscape change, Mountain agriculture, Central Andes, Peru

## Abstract

Policy makers concerned with the peri-urban interface find their greatest challenges in the rapid urban growth of developing mountain regions, since limitations caused by relief and altitude often lead to an increased competition between rural and urban land use at the valley floors. In this context, little attention has been paid to the affected agriculturalists’ perceptions of peri-urban growth—important information required for the realization of sustainable land use planning. How is the process of rural–urban land change perceived and assessed by peri-urban smallholder communities? Which are the major difficulties to be overcome? By what means are the affected people reacting and how are these adaptation strategies linked with the ongoing landscape transformations of the hinterland?

By using the example of Huancayo Metropolitano, an emerging Peruvian mountain city, it is shown that rural–urban land change is intensively discussed within peri-urban smallholder groups. Although urbanization also leads to infrastructure investments by public institutions—an advantage perceived throughout the study area—the negative impacts of rural–urban land use change prevail. The perceptions’ analysis reveals that the decrease of fertile and irrigated agricultural land at the quechua valley floor is especially considered to threaten subsistence, food and income security. In order to compensate the loss of production capacities, many smallholders try to expand or intensify their land use at the suni altitudinal belt: an agro-ecological zone characterized by steep and nonirrigated slopes that can actually not be used for the year-round production of crops previously cultivated at the quechua zone.

## Highlights

• A set of 20 perceptions toward rural–urban land change is inductively determined.
• Six major impacts of urbanization on peri-urban smallholders are identified.
• Service and infrastructure improvements are highlighted by more modernist groups.
• Urbanization is negatively perceived by traditionalist smallholder communities.
• Perceived income insecurity leads to adaptation of land use on steep slopes.

## Introduction

### Background and aims

Due to the socio-economic and biophysical diversity of hybrid rural–urban areas, the highly dynamic land use patchwork at the peri-urban^1^ interface (Allen, 2003; Brinkley, 2012; Qviström and Cadieux, 2012; Simon, 2008) has received increasing attention by applied research, land use planning and policy making—especially over the last decade. To a certain degree, this tendency also applies to studies on the perception of land use change. Gilg (2009) concludes that urban areas are often grossly overestimated by farmers and that the “rural idyll” remains a persistent myth within urban populations; recent studies (Ives and Kendal, 2013; Slemp et al., 2012; Soini et al., 2012; Swanwick, 2009) moreover show how changing peri-urban landscapes are perceived by different stakeholders. Research in this area, however, has predominantly been carried out in more developed countries and has hardly considered regional geographic specificities.

Mountain valleys, for instance, represent a peculiar type of space that is particularly vulnerable to urban sprawl and rural–urban land use change, as the construction of roads and thus settlement expansion occurs mainly on the arable land of the valley floors and adjoining lower slopes. Consequently, rapid urbanization—especially if of low density—causes both changes in the use of environmental resources (such as land for food production) and social transformations within the hinterland's rural communities. MacDonald and Rudel (2005) have underlined the peculiar patterns of residential and forest land use in New Jersey's exurban Appalachian valleys and, in this context, Rudel et al. (2011) have pointed out that the impacts of the recently emerging, more exclusive forms of land use on society and environment remain poorly understood. Also with respect to the European Alps, several studies on urbanization and the land use developments mentioned have already been carried out (Bertrand and Vanpeene-Bruhier, 2007; Hersperger and Bürgi, 2009; Perlik et al., 2001; Peyrache-Gadeau and Fleury, 2005). Regarding the Central Andes, by contrast, in recent years less attention has been paid to the rural–urban restructuration and its perception by local people—especially in the hinterland of intermediate mountain cities. Yet, this is eminently necessary, as many medium-sized Latin American cities (Bolay and Rabinovich, 2004; Goluchowska, 2002; Klaufus, 2012; Stadel, 2001; Steel, 2013) are following the metropolises’ development of major, globalization-driven urban restructuring (Borsdorf and Hidalgo, 2008, 2013; Portes and Roberts, 2005; Roberts, 2005); different sociocultural attitudes (Knapp, 2010), mainly neoliberal versus indigenous *Weltanschauungen*, lead to varying understandings of how land use should be steered by planners and policy makers (Othengrafen and Reimer, 2013). Moreover, rising socio-ecological inequalities within the rural–urban mountain landscape—a tendency partially boosted by poverty-driven rural–urban migration and lifestyle-oriented urban–rural movements—conspicuously entail potential for land use conflicts between the stakeholders. Among the respective groups of interest, the inhabitants of peri-urban agrarian settlements are often not taken into account in urban planning, probably because the rural vernacular is not considered part of the modern city. However, these people represent a group directly affected by urban sprawl; albeit not always detrimentally (Robinson, 2008, p. 25; Satterthwaite et al., 2010).

Given the UN-HABITAT program's objectives of participatory problem solving and propoor governance, the present case study generally aimed at investigating the locals’ perceptions of ongoing rural–urban land changes, and thus supporting decision making for sustainable development and management in the hinterlands (Raymond et al., 2010) of Andean mountain cities. Using the example of Huancayo Metropolitano, a Peruvian mountain city of currently 420,000 inhabitants distributed over seven districts (Haller and Borsdorf, 2013), the study's specific aims were as follows: (1) to determine the smallholders’ attitudes toward the urban sprawl of Huancayo Metropolitano; (2) to understand the consequences of urbanization for their land use; (3) to interpret these assessments against Haller's (2012) quantitative results of Huancayo's rural–urban land change. What are the impacts of urbanization on the smallholder livelihoods perceived by the affected communities themselves? Are they profiting from this residential development by selling lots to the new peri-urban dwellers? What further consequences does it have for the agricultural land use and how may these changes be linked to the Andean landscape transformation?

Temporally, the focus was laid on the last 15 years (1998–2013), for the *Zeitgeist* of neoliberal policies in Peru arose with ex-president Alberto Fujimori during the 1990s. Epistemologically, the present research was mainly positioned between empiricism and humanistic perspectives, and included a historicist vision (Gade, 2011; Rudel, 2009); by applying the inductive method, observation generally allowed a step-by-step approximation from individual cases to a characteristic type of perception, while hermeneutic interpretation additionally led to a better understanding of these attitudes’ impact on the cultural landscape's genesis.

### Study area

**Fig. 1:**
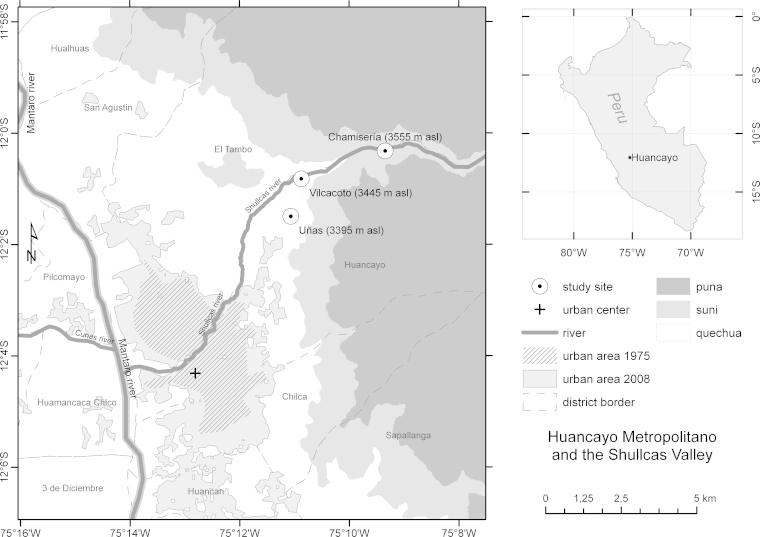
The case study sites in the lower Shullcas Valley near Huancayo Metropolitano. The continuous urban area's growth 1975–2008 is shown. The map has been elaborated on the basis of 1975 Landsat 2 MSS, 2008 Landsat 5 TM and Aster GDEM data.

The Central Peruvian agglomeration of Huancayo Metropolitano (3260 m asl), situated at approximately 12°4′S and 75°12′W between the Western and Eastern Cordillera in the Mantaro Valley, has changed from a rural town of around 6000 people (at the end of the 19th century) to an emerging commercial agglomeration that is now undergoing major socio-economic changes (Haller and Borsdorf, 2013; Roberts, 1995). Its urban center is located on the alluvial fan of the Shullcas River, a tributary of the Mantaro River that issues near the Chuspicocha (from Quechua *ch’uspi* for “fly” and *qocha* for “lake”) and Lasuntay—from Quechua *qasa* for “frost” and *quntay* for “smoke” (Cerrón-Palomino, 1989)—glacial lakes at 4600 m asl. For the purposes of the present qualitative research, the orographic left side of the lower Shullcas Valley (Fig. 1), which is entirely situated within the district of Huancayo, has been considered eminently suitable: (1) it represents a zone of major peri-urban development in Huancayo Metropolitano (Haller and Borsdorf, 2013) and is locally known as one of the city's best residential areas; (2) it is mainly situated below 3500 m asl within the *quechua* altitudinal belt (Pulgar Vidal, 1946; Zimmerer and Bell, 2013)—the zone where almost all urbanization processes occur (Haller, 2012).

Direct field observations have identified Palián to be the most peripheral of the fully urbanized settlements in the Shullcas Valley. This limit coincides with the end of the “zone for district commerce” (*zona de comercio distrital*) and the beginning of the “low density residential zone” (*zona residencial de densidad baja*), as defined by the municipality's urban land use zonation 1996–2005 (Municipalidad Provincial de Huancayo, 1996). Consequently—using the 1993 statistics of hamlets and villages (Instituto Nacional de Estadística e Informática, 1993) as well as a topographic map (scale 1:100,000) of the National Geographic Institute (Instituto Geográfico Nacional, 1999)—all the district's statistically defined agrarian settlements or *unidades agropecuarias* located beyond Palián and within the *quechua* altitudinal zone have been taken into account: Uñas, Vilcacoto and Chamisería; while the first one lies within the “low density residential zone”, the latter are already part of the “inviolable agricultural zone” (*zona agrícola intangible*). The individuals belonging to the agrarian villages’ families have then been considered smallholders, even though not all family members are working in the primary sector. Following (Figueroa, 1984, pp. 13–14), these mostly nuclear families are defined as consisting of those persons who are living in the same house.

## Materials and methods

### Sampling design

Given the study's research design (audio-recorded, structured interviews exclusively carried out by the author), the desired sample size of 75 persons, the study area's spatial extent (approximately 4000 m × 500 m) and the time exclusively available for interviews (one month), nonprobability quota sampling appeared best suitable for the planned qualitative research process.

This method aims at achieving a sample structure similar to that of the total population (often known through census data; Table 1)—for example regarding the relative distribution by gender within a certain area—in order to allow a certain degree of generalization (Daniel, 2012, pp. 105–107) and is mostly applied if no list of the statistical population's elements exists. On the one hand, for the interviewees are not randomly selected, the sampling error cannot be estimated and the selection bias is not minimized. On the other hand, however, the consideration of the variables of interest (place of residence, gender, age) within a proportional quota sampling plan increases the probability to include even elements of small groups and enabled a more detailed identification of perceptions within the sample.

Since the statistical information shown in Table 1 only offered separate data (gender, age) about the respective settlements’ population—without giving details on gender ratios per age group—two assumptions were made: (1) the female surplus was rather a product of male outmigration than the result of a larger number of female births; (2) outmigration for labor or higher education occurred predominantly within the second age group (greater than 14 years). Thus, an equal sexual proportion was supposed for the first segment (less than or equal to 14 years). Consequently, in order to maintain the overall female surplus (Table 1), the more realistic combined ratios among the elder population resulted as shown in Table 2.

The latter strata (males and females >14 years) were then considered the only relevant, as younger persons were not supposed to make land use decisions. Moreover, it was assumed that the relative distributions of the variables of interest from 1993 would be valid for the 2013 situation. Thus, by multiplying the target group's percentages per place (Table 2) by the respective settlement's total population number—and rounding the results to integer values—the combined quota sampling plan (sample size of 75 interviewees) could be defined (Table 3).

### Interview design

In view of the study's aims and research questions, a structured interview design was chosen for screening perceived impacts of rural–urban land change on peri-urban smallholder farmers. Due to the interviews’ short duration (up to 15 minutes each), the number of noncooperative potential interviewees was very low. Thus, this technique permitted to personally interview a relatively large number of people, as well as to categorize and compare different perceptions among the target population. For the results of these exploratory interviews were well codeable, they were reasonably interpretable—an important fact if the research's methodology and output should contribute to the design of future sustainable land use policies. The focus was rather laid on the responses’ content than on their quantitative aspects; hence, its design was broadly of qualitative character. All interviews were audio-recorded; while the respondents’ narrative answers to the two open questions were subsequently transcribed, the response to the final closed question on the overall assessment of rural–urban transition was directly marked by the interviewer on the questionnaire.

In order to select appropriate interviewees, people were asked whether they had their place of residence in the study area at least during the last 15 years, for this was a key characteristic. Next, the questionnaire was primarily structured along this study's research questions. Some introductory words (one minute) were followed by two separate open questions (5 minutes each), where the interviewees were asked to talk about both the positive and the negative impacts of peri-urban growth during 1998–2013 along the lower Shullcas Valley on the local smallholders’ life:

“In your opinion, what were the {advantages, disadvantages} of the city's urban expansion toward {Uñas, Vilcacoto, Chamisería} for the local smallholders’ life during the last 15 years?”

Additionally, the respondents were queried whether the positive or the negative impacts of urban sprawl—or none of both—prevailed (2 min). Moreover, approximately two additional minutes were kept free for short, conversations on the study site's landscape change during 1998–2013, as well as for some personal questions at the end of each interview.

### Data analysis and interpretation

The recorded interviews held in Spanish were afterward transcribed using conventional word processing software. Thereby the interviewer's questions were excluded, for they were identically asked within each interview. Further, the transcripts did not include information about paralanguage, as this type of content was not considered important; regarding orthography, the transcription followed the standard rules of the Real Academia Española. Next, a qualitative analysis of the manifest content (Hsieh and Shannon, 2005; Krippendorff, 2013; Mayring, 2000; Schreier, 2012)—the visible, obvious text components—was applied in order to systematically classify the perceptions and to enable an intersubjectively understandable interpretation. Methodologically, an inductive process, similar to those described by Hällgren Graneheim and Lundman (2004) or Elo and Kyngäs (2008), was adopted for the present exploratory study's purposes (Table 4). The segmentation of the respective answers (the units of analysis) into meaning units—and a summarization of the latter (condensation; a description close to the text)—enabled the responses’ comparison and thus the generation of reasonable subcategories (abstraction). Thus, the subcategories emerged “bottom up” (inductively) out of the data. Finally, the latter subcategories were analyzed and could be interpreted against the asked research questions (the predefined main categories “advantages” and “disadvantages”) in order to better understand the existing perceptions.

## Results and discussion

The 76 interviews conducted in February 2013—thus in the agriculturally active rainy season—comprised 37 persons in Uñas (17 male, 20 female), 34 in Vilcacoto (16 male, 18 female) and five in Chamisería (two male, three female); hence, the requirements of the previously designed quota sampling plan could be considered fulfilled.

### Pros and cons of rural–urban land change

The 20 subcategories, which represent either perceived advantages or disadvantages of rural–urban land change for the peri-urban smallholders, show that the diversity of negative impacts (15) is clearly higher than the variety of positive impacts (five). Thereby, the number of subcategories (Table 5) that may be summarized as belonging to a group of socio-economic consequences (identifiers A, B, F, G, I, J, K, L, N, R and S) is insignificantly higher than that of subcategories assignable to a group of socio-ecological impacts (C, D, E, H, M, O, P, Q, T). This equal distribution is probably due to the close human–environment linkages that are typical for the rural Central Andean realm (Gade, 1999)—where the societal awareness of the natural resources’ importance for life-sustaining economic activities (e.g. food production) is traditionally high.

**Fig. 2:**
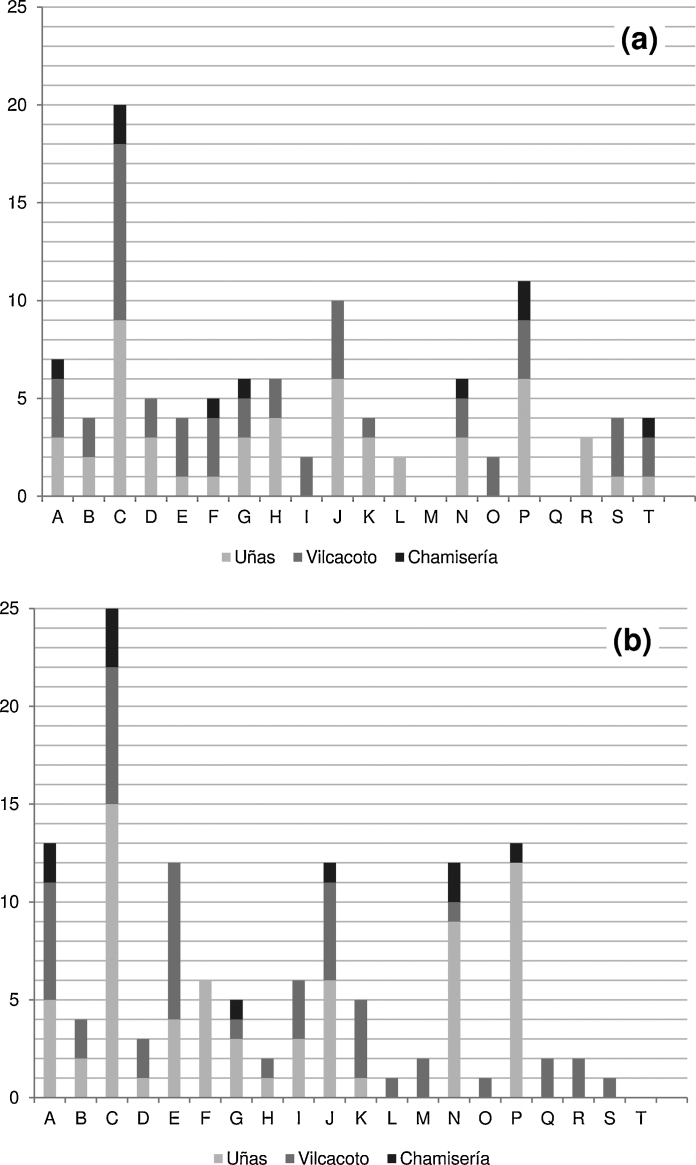
Absolute frequency values of the subcategories’ appearance by place for male (a) and female (b) interviewees.

The socio-economic perceptions draw attention to the urbanization-driven changes within the agrarian communities; outmigration of smallholders as well as the inmigration of people of cultural attitudes contrary to those of the locals lead to the weakening or partial destruction of social networks. Contrary to the assumption that the migration of Huancayo's amenity-seeking upper class to the peri-urban interface would cause discomfort for the agrarian settlements’ inhabitants, the arrival of other smallholders from the higher *suni* (3500–3800 m asl) and the adjoining *puna* (3800–5200 m asl) altitudinal zones, which migrate to the rural–urban fringe at the *quechua* level in search of new opportunities, is more often perceived as disadvantage, for these “uneducated people” would not respect the local environment and society (e.g. by contaminating the Shullcas River). With respect to the negative socio-economic impacts of urban origin, the increasing delinquency and drug abuse—often perceived together with the appearance of *gente de mal vivir* (literally “evil-living people”)—is frequently mentioned. Yet, the increase of the settlements’ population numbers per se is seen as an advantage of rural–urban land change, since the inhabitants feel that this demographic dynamic leads to a greater attention paid to the villages by private entrepreneurs and Huancayo's public policy makers; thereby, the improvements in infrastructure services (water management, transport, communication) and the additional commercial possibilities are perceived as advantages of great significance for the smallholders’ daily life. Regarding the subcategories’ absolute frequency values by gender it is shown (Fig. 2) that C (“caused the loss of agricultural land”), J (“entailed the improvement of transport”) and P (“forced people to cultivate high altitude land”) stand out within both male and female results. Women, however, additionally highlight the effects of rural–urban land change on commercial possibilities (A), land and water contamination (E) and subsistence, food and income security (N). The latter three perceptions might be explained by the multiple roles women play in rural families, while male smallholders are predominantly engaged in agriculture. In sum, the relatively low average number of subcategories per interview (between three and four) indicates that those few thematic areas mentioned by the smallholders are clearly dominating their respective attitudes toward urban expansion and thus play a decisive role for them.

Closely related to the negative socio-economic impact on subsistence, food and income security, the effects of rural–urban land use transitions on the landscape setting at the valley floor (loss of agricultural land) seem to be greatly feared by the smallholders; according to some interviewees, most smallholders only own small parcels of land (*minifundios*), which are used for subsistence farming. Moreover, many of them perceive that wealthy and well-educated land investors from outside cheat the humble *minifundistas*, seducing them into selling lots—at prices significantly below the market value. Furthermore, many of the often cash-poor smallholders rent additional parcels of arable land in the agriculturally favorable *quechua* zone, in order to grow crops of high market demand—such as corn (*Zea mays*), artichokes (*Cynara cardunculus*) or potatoes (*Solanum* spp.)—for sale; consequently, the positively perceived monetary valorization of land seems to be rather a burden than a benefit. In sum, the statements of a breeder of guinea pigs from Vilcacoto—representative for many other urbanization-critical interviewees—describe best the negative perceptions of rural–urban land change smallholders have:

“The problem is the incrementing number of houses; these dwellers are sowing concrete! I call it the sowing of concrete. There, on the fertile and productive land, they are sowing concrete. In future there will not be any food production! […] There are owners—peasants—who once cultivated their land; but now they take the easy way out, selling their land, migrating to the city, buying a car. They care little about the production of food.”

This comment clearly indicates the differing points of view existent within the smallholder population. One group—the more traditionalist—claims the protection of arable land in order to safeguard the food production for themselves and for future generations. Members of the opposite group, by contrast, consider Andean agriculture to be backward, and evidently prefer their villages’ integration into the “civilized” urban world; often justifying rural–urban land change by using the mainly meaningless connotations “progress”, “advancement” or “development”, as the opinion of an evangelical Protestant Christian from Vilcacoto exemplifies:

“Other smallholders have to go up to high altitude areas—or even migrate down to the rain forest; but you know: development is progress and a man who serves is a living man—one who does not is a dead man! […] Serving to the humanity is progress, or is it not? Hence, those who migrate for working away from home afterward bring money in order to continue in their villages.”

For understanding the consequences of rural–urban land change for the smallholders’ land use, it is helpful to determine those of the most wide-spread impact perceptions that have the greatest perceived advantages or disadvantages. Therefore, all subcategories (Table 5) that appear within at least five of the six groups of interest are selected. Next, it is assumed that those advantages (respectively disadvantages), which are—in relative terms—most frequently mentioned in combination with a positive (respectively negative) total evaluation of urban expansion, are of greater effect for the smallholders’ land use decisions.

**Fig. 3:**
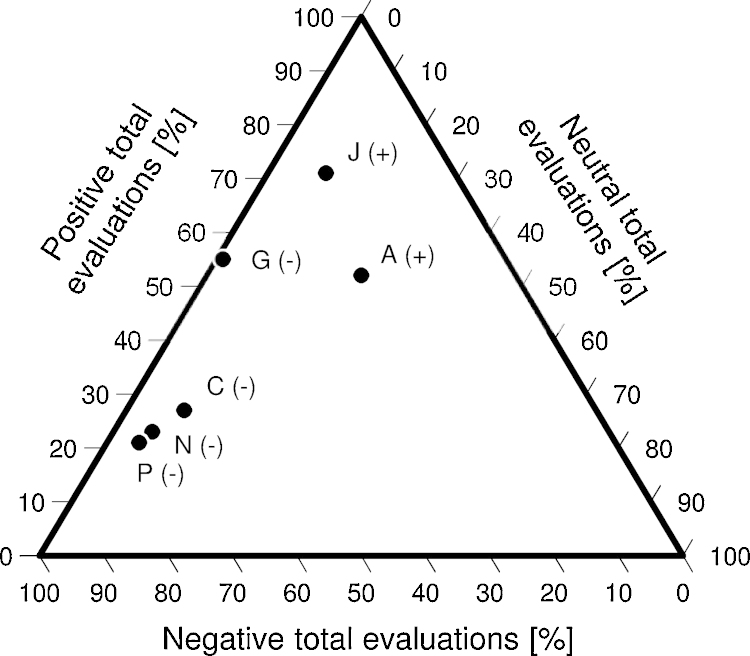
The subcategories A (*n* = 20), C (*n* = 45), G (*n* = 11), J (*n* = 22), N (*n* = 18) and P (*n* = 24); showing the relative results of the total evaluation regarding the total number of those interviews whose answers contained the respective subcategory.

**Fig. 4:**
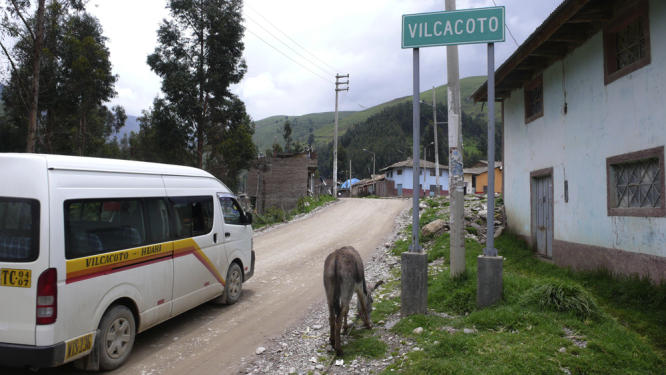
Donkeys are overtaken by minibuses. New transport infrastructure is considered a positive impact of rural–urban land change by smallholders in Vilcacoto.

As shown in Fig. 3—a simple form of a triangular diagram designed using a Microsoft^®^ Excel spreadsheet developed by Graham and Midgley (2000)—, the disadvantages C (“caused loss of agricultural land”), N (“affected subsistence, food and income security”) and P (“forced people to cultivate high altitude land”) are mentioned in combination with a negative total evaluation in approximately 70% of the respective cases, whereas the increased outmigration of smallholders (identifier G) seems not to be such a strong argument for a negative total appraisal (45%); although it is considered a disadvantage, the smallholders are aware of this process’ positive impacts, for the outmigration of others leads to the remittance of *capitalcito* (literally “some money”) and thus contributes to the villages’ “progress”. With respect to the advantages of rural–urban land change, the improvement of transport (Fig. 4) represents the subcategory that most often coincides with a positive overall rating (70%) within the respective interviews; the urbanization-driven creation of new commercial opportunities, by contrast, shows little more than a coincidence of 50%.

### Land use change and verticality

Regarding these perceptions’ effects for the smallholders’ land use, the interplay between the perceived disadvantages C, N and P merits a deeper analysis. For the demand of land at the urban margin is rising—driven by the amenity-seeking urban and the rural poor—and the availability of land at the valley floor is being decreased due to soil sealing, the smallholders’ possibilities to rent additional land for the production of crops grown for sale (corn, artichokes, potatoes) are reducing. Many smallholders then react by expanding or intensifying land use at the higher *suni* and *puna* altitudinal belts, where the land predominantly belongs to the agrarian communities. These perceptions and their consequences for land cover match with the quantitative results presented by Haller (2012), who—basing on remote sensing and GIS analyses—reports the expansion and intensification of forestry and agriculture at the *suni* and *puna* of Huancayo's hinterland.

Yet, this adaptation strategy implicates several further disadvantages for the affected agriculturalists: (1) the now improved road infrastructure does not reach up to the high altitude fields and, thus, no longer represents an advantage for the transportation of goods; (2) the perceived possibilities for growing cash crops are reduced, since corn and artichokes cannot be successfully cultivated above the *quechua* zone; (3) the year-round production of potato is hardly possible at the higher altitudinal belts, as there is—contrary to the valley floor—a lack of irrigation infrastructure. Consequently, the agricultural activities are limited to the rainy season, as a *comunero* (community member) from the San Vicente association explains:

“Here in our community San Vicente, some have very small parcels. They only sow for their own consumption—nothing more, nothing for sale. Up there, on the steep slopes there is communal land, which is divided between the *comuneros*; there we could sow more—but this land is not irrigated! We eagerly await the rain, then we sow—without rain, by contrast, there is nothing! The municipality and the regional government do not support us at all!”

These conclusions underline the peri-urban smallholders’ dilemma; while Zimmerer (1999) reports that areas used for irrigated and market-oriented agriculture expand in the relatively remote rural Paucartambo Valley (from Quechua *pawqar* for “flowery” and *tampu* for “lodge”)—due to the rising demand of potatoes by the growing urban society in Cusco—the peri-urban smallholders in the Shullcas Valley lose many of these irrigated fields owing to rural–urban land change. For an evangelical inhabitant of Vilcacoto, these processes are even reminiscent of descriptions known from Christianity (“Urbanization will cause a long drought; as it stands in the Bible!”).

**Fig. 5:**
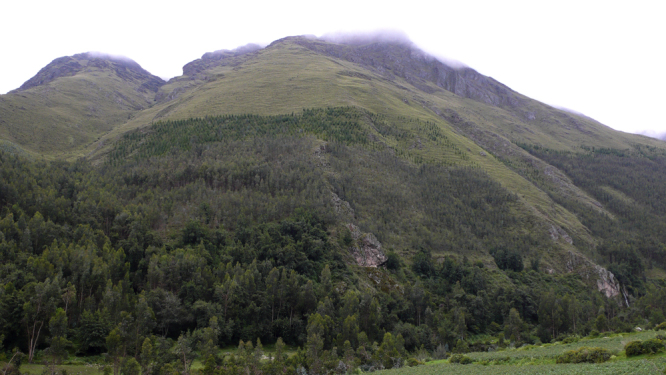
In the hinterland of Huancayo (Shullcas Valley), the *suni* altitudinal zone is increasingly covered by *Eucalyptus globulus* and used for the production of wood. The upper line of eucalyptus plantations in the photo (taken in February 2013) is at 3700 m asl.

For the other traditional tubers of the *suni* such as the high-yielding mashua (*Tropaeolum tuberosum*), the hardy and frost-resistant oca (*Oxalis tuberosa*) and the moderately drought-resistant olluco (*Ullucus tuberosus*) are not perceived to be of high market potential, more and more smallholders opt for the plantation of *Eucalyptus globulus* (Fig. 5) in order to compensate the income sources lost through urbanization. This strategy is obviously a product of the growing demand for wood by the construction sector—a branch that clearly profits from rural–urban land change in the *quechua* region. Haller (2012) estimated a plus of 114% (during 1988–2008) regarding the land covered by trees and shrubs at the *suni* level. Thereby, the results of direct field observations in 2011 and 2013 indicate that large parts of this land cover change are linked to the cultivation of the wood crop mentioned. Against these backgrounds, one could hypothesize that, in times of Andean urbanization, the steep, nonirrigated slopes of the *suni* hinterland are becoming the peri-urban smallholder communities’ new areas for market-oriented production.

In sum, it can be stated that the land market's dynamics at the valley floor are main drivers of the smallholders’ land use expansion or intensification at the higher altitudinal belts of the city's hinterland. Surprisingly, exactly this market could have been stimulated by Juan Velasco's—then president—agrarian reform during the 1970s (Mayer, 2009). As Calderón Cockburn (2006) illustrated for the case of Lima, many large land owners who feared the expropriation converted themselves into real estate developers and sold their lots for urbanization.

### Protection for production?

Although not explicitly asked, many interviewees have expressed their visions about how the municipality's future land use policy should look like. As exemplified by the ideas of an elder female smallholder from Uñas, many respondents underline the need for farmland conservation along the Shullcas Valley:

“From my point of view, this area once was a beautiful agricultural landscape, where the people harvested potatoes, corn, beans—all types of food! I do not agree at all [with the process of urbanization]; today, there is not any production—but there is concrete! Yes, this development of course affects me. Apart, the new dwellers construct tiny houses; they do not think about the future! There should be three-storied buildings; we need more planning! Although urbanization brings us more civilization, in my opinion the agricultural land should be protected. […] Otherwise, Huancayo will not have any food production anymore. Then, what will we eat? We would have to buy everything from other provinces!”

Surprisingly, some smallholders demand the construction of multi-storied buildings, while others propose the construction of houses on the steep slopes—in order to protect the fertile alluvial soils of the valley floor. Thereby, a female Vilcacoto inhabitant's point of view emphasizes that the more traditionalist smallholders’ claims for farmland protection are rather motivated by the fear of losing food and income security than by the desire for esthetic landscapes:

“What will we eat now—houses? It would be better to urbanize the hills instead of the fertile agricultural land; because our life is based on the cultivation of crops. This development threats us. Without a doubt urban growth also has a positive side; yet, our agricultural production should not be damaged. […] Thus, urbanization severely affects us, it is completely negative.”

The protection of *quechua* farmland by using a conventional zoning approach (e.g. Euclidian zoning), however, seems not to be a solution for the challenges perceived by the smallholders! A simple comparison of the in situ situation 2013 with the urban land use zonation plan 1996–2005 (Municipalidad Provincial de Huancayo, 1996) makes clear that the so-called “inviolable agricultural zone” (*zona agrícola intangible*) is either inexistent or has been easily changed in the past. Against the background of often unclear ownership structures, decreasing supply and increasing demands of lots, the already mentioned dynamics of the land market at the *quechua* level (the zone best suitable for settlement) hinder the practical realization and supervision of conventional top down zoning policies. In this context, the establishment of “meta zones” (zones of different planning policies) could best represent a planning approach that takes the fragmented patchworks of agriculture and settlement on the Andean cities’ outskirts (Borsdorf, 2003; Zimmerer, 1999) into account; while the fully urbanized parts of the city would represent an area suitable for traditional Euclidian zoning, performance zoning (Baker et al., 2006; Cools et al., 2002; Ottensman, 2005) could be convenient for the highly dynamic peri-urban interface of the *quechua* altitudinal belt. Consequently, the latter “meta zone” must be planned using methods that define priority areas for conservation (Garrard et al., 2012; Orsi and Geneletti, 2010). Thereby, an initial investigation of existing environmental perceptions of different stakeholders—for defining a common baseline—would probably help to “calibrate” or align the planners’ and policy makers’ attitudes with the assessments of the affected smallholders.

Furthermore, serious policy efforts to foster the peri-urban smallholders’ resilience (Coy, 2010; Stadel, 2008) to the perceived food and income insecurity should focus on the use of the rural hinterland's verticality and ecological complementarity *sensu* Murra (2009, pp. 83–142). The altitudinal zones of the *suni* and *puna* would offer abundant possibilities for the production of fresh food crops, their processing—e.g. *toqosh* (fermented potato pulp) or *chuño* (freeze-dried potatoes)—and patrimonialization (Gade, 2004). This would bear the potential to link both the valorization of cultural landscapes and the production of traditional food with the urban-based consumers, who still perceive the importance of native crops such as maca (*Lepidium meyenii*) or quinoa (*Chenopodium quinoa*) for the local identity (Córdova Aguilar et al., 2005). Such a conservation-with-intensification approach (Zimmerer, 2013), however, would further require a vitalization of the smallholder–market linkages (Rist, 2000). Examples from rural Alpine (Bender, 2010) and pre-Pyrenaic metropolitan cases (Paül and McKenzie, 2013) prove the idea's feasibility in different geographic settings.

## Synthesis and outlook

The present research results show that rural–urban land change is a phenomenon clearly perceived by the study sites’ peri-urban smallholders; the wide range of 20 socio-economic and socio-ecological impacts mentioned—be they positive or negative—prove the importance Central Andean smallholders ascribe to the processes of urban expansion. Moreover, by example of the most negatively seen disadvantages, the analysis explains the strong interrelations between these impacts and, consequently, their effects on the smallholders’ land use behavior. In this context, the methodological approach applied proves the usefulness of analyzing in situ-gathered primary data on environmental perceptions by using both quantitative and qualitative techniques—a strategy that doubtlessly challenges the researcher to shift between the objectivist (empiricist) and the more subjectivist (humanist) position.

The perceptions of rural–urban land change, determined via structured but mainly open-ended interview questions, reveal that a single homogenous group of smallholders no longer exists; cultural influences evidently have divided the villages’ communities into more indigenous-oriented traditionalist (tend to be urbanization opponents) and neoliberal modernist groups (rather proponents). Both are aware of pros and cons—nevertheless, the more modernist minority stresses the positive effects of population growth on both the government's infrastructure investments and the growing commercial potential, while the numerous traditionalists especially perceive negative impacts related to food and income insecurity; yet, the quoted comments as well as the land use decisions driven by the latter perception convey the impression that—more than food shortages—the loss of monetary income is feared by the peri-urban smallholders. The *minifundistas* feel that, due to rapid urban growth, their possibilities to rent additional parcels for growing food crops are dramatically decreasing. In order to compensate the irrigated and market-oriented food production lost, more and more steep, nonirrigated slopes of the *suni* are reforested with wood crops that enjoy high market demand, and, to a lower extent, used to cultivate potatoes during the rainy season.

In sum, the regional focus on geographic peculiarities of coupled human–environmental systems in the area to be planned—such as the Central Andes (Borsdorf and Stadel, 2013)—has demonstrated that future *ex-ante* and *ex-post* appraisals of land use changes should concentrate on the affected local people's perceptions, for this would bridge the social gap between the “well-educated” planners’ real world and the “humble” smallholders’ perceived environment; it thus could help to reach the UN-HABITAT program's objectives of participatory problem solving and propoor governance. Especially in times of neoliberal urban planning policies (Sager, 2011), a more humanist attitude on behalf of the local authorities and real estate developers (including the empathy necessary for recognizing the smallholders’ perceived experiences of urbanization) would already represent a step forward toward sustainable—perhaps performance-based?—land use planning in peri-urban environments. Hence, on the basis of a deeper, mutual understanding between peri-urban agriculturalist families and land use policy makers, the “sowing of concrete” might result in a bountiful harvest for both sides—the UN International Year of Family Farming 2014 would represent a perfect starting point.

## Figures and Tables

**Table 1:** Population characteristics of the study sites in 1993.

Place name	Quantification	Gender		Age	
		Male	Female	≤14 years	>14 years
Uñas	Absolute [count]	436	485	413	508
	Relative [%]	47.3	52.7	44.8	55.2

Vilcacoto	Absolute [count]	426	499	432	493
	Relative [%]	46.1	53.9	46.7	53.3

Chamisería	Absolute [count]	66	72	68	70
	Relative [%]	47.8	52.2	49.3	50.7

Total	Absolute [count]	928	1056	913	1071
	Relative [%]	46.8	53.2	46.0	54.0

**Table 2:** Age groups and their assumed shares of the respective settlements’ population by gender.

Place name	Gender	Age	
		≤14 years	>14 years
Uñas	Male [%]	22.5	25.0
	Female [%]	22.5	30.0

Vilcacoto	Male [%]	23.5	23.0
	Female [%]	23.5	30.0

Chamisería	Male [%]	24.5	23.5
	Female [%]	24.5	27.5

Total	Male [%]	23.0	24.0
	Female [%]	23.0	30.0

**Table 3:** The quota sampling plan: relative shares refer to the target group's 1993 population.

Place name	Gender	Age >14 years		Quota [persons]
		Absolute [count]	Relative [%]	
Uñas	Male	231	21.6	16
	Female	277	25.9	20

Vilcacoto	Male	213	19.9	14
	Female	278	26.0	20

Chamisería	Male	33	3.1	2
	Female	39	3.6	3

Total		1071	100.0	75

**Table 4:** The categorization workflow by step (1 denotes the first task), process and result.

Step	Process	Result	Explanation	Example
1	Predefinition	Main category	The predefined research questions determine the main categories	Advantages of urban expansion for the peri-urban smallholders’ life
2	Segmentation	Original meaning unit	The transcribed interview results are divided into meaning units	Urban expansion is good for the village, which now is advancing; for example the road has been enlarged, and so on.
3	Condensation	Condensed meaning unit	Each original meaning unit is summarized and simplified	Urban expansion has caused improvements of the road.
4	Abstraction	Subcategory	Abstracted categories are created for similar content	Entailed the improvement of transport

**Table 5:** Appearance (1) and nonappearance (0) of perceived advantages (+) and disadvantages (−) of rural–urban land change; displayed by place of residence and gender. The subcategories emerged out of the transcribed interviews by segmentation, condensation and abstraction.

ID	Subcategory	Uñas		Vilcacoto		Chamisería		Sum
		Male	Female	Male	Female	Male	Female	
A	Created new opportunities for commerce (+)	1	1	1	1	1	1	6
B	Led to the arrival of evil-living people (−)	1	1	1	1	0	0	4
C	Caused the loss of agricultural land (−)	1	1	1	1	1	1	6
D	Drove the destruction of wood and shrubland (−)	1	1	1	1	0	0	4
E	Resulted in contamination of land and water (−)	1	1	1	1	0	0	4
F	Seduced smallholders into selling lots (−)	1	1	1	0	1	0	4
G	Increased outmigration of smallholders (−)	1	1	1	1	1	1	6
H	Conducted to a better water management (+)	1	1	1	1	0	0	4
I	Raised inmigration of uneducated people (−)	0	1	1	1	0	0	3
J	Entailed the improvement of transport (+)	1	1	1	1	0	1	5
K	Boosted delinquency and drug abuse (−)	1	1	1	1	0	0	4
L	Generated egoism and competition (−)	1	0	0	1	0	0	2
M	Impaired the smallholders’ health situation (−)	0	0	0	1	0	0	1
N	Affected subsistence, food and income security (−)	1	1	1	1	1	1	6
O	Contributed to the valorization of lots (+)	0	0	1	1	0	0	2
P	Forced people to cultivate high altitude land (−)	1	1	1	0	1	1	5
Q	Induced air pollution in the village (−)	0	0	0	1	0	0	1
R	Necessitated the use of fertilizers (−)	1	0	0	1	0	0	2
S	Brought communication technology (+)	1	0	1	1	0	0	3
T	Produced biological resources scarcity and loss (−)	1	0	1	0	1	0	3

## Footnotes

1 In the present study, the term “peri-urban” is used in a double sense (thus meaning “the marginally urban at the urban margin”). I refer to those parts of the formerly exclusively rural hinterland that are now characterized by mixed rural and urban land use and land cover structures. Peri-urbanization (Zasada et al., 2011, p. 63) is thus that part of “rural–urban land change” (interchangeably used with “urbanization”) that temporarily or permanently leads to peri-urban landscape structures.
